# Editorial: Translational advances in cardiovascular therapy: from bench to bedside

**DOI:** 10.3389/fcvm.2025.1662309

**Published:** 2025-07-29

**Authors:** Sang-Bing Ong, Ming-Tao Zhao, Lei Ye

**Affiliations:** ^1^ Department of Medicine and Therapeutics, Faculty of Medicine, The Chinese University of Hong Kong, Hong Kong, Hong Kong SAR, China; ^2^ Center for Cardiovascular Research and the Heart Center, Nationwide Children’s Hospital, Columbus, OH, United States; ^3^ Department of Pediatrics, The Ohio State University College of Medicine, Columbus, OH, United States; ^4^ Department of Biomedical Engineering, School of Medicine and School of Engineering, The University of Alabama at Birmingham, Birmingham, AL, United States

**Keywords:** cardiovascular diseases, heart failure, nanoparticles, arrhythmia, clinical investigation

**Editorial on the Research Topic**
Translational advances in cardiovascular therapy: from bench to bedside

Cardiovascular diseases (CVDs) are the leading cause of mortality worldwide. About 17.9 million people died from CVDs in 2019, which represent 32% of all global deaths. In recent years, advances in molecular and cellular biology, especially genetics, stem cell biology, and developmental biology are transforming the way we understand and treat heart failure.

In this Research Topic, entitled “*Translational Advances in Cardiovascular Therapy: From Bench to Bedside*”, six original research papers depict some underlying mechanisms precipitating in cardiac disorders and modulatory measures to mitigate the dysfunction of the myocardium. The studies include an understanding of zinc oxide nanoparticles (ZnO NPs) in induction of arrythmia, how monocyte adhesion to and transmigration through endothelium, immunomodulation role of CD16^+^ monocyte in myocardial infarction, and the association between iron metabolism, transferrin saturation level, and stress hyperglycemia ratio with cardiac diseases.

Liu et al., explored the effect and underlying mechanism of ZnO NPs exposure on cardiac function, especially during acute exposure. The study showed that acute exposure to ZnO NPs markedly decreased voltage-gated sodium current (I_Na_) and long-lasting calcium current (I_ca−L_), resulting in a reduced amplitude and shortened action potential duration in cardiomyocytes. These changes not only prolonged PR-interval and blocked A-V conduction that triggered cardiac arrhythmia, but also led to a diminished calcium transient, which contributed to heart failure in a mouse model. The downregulation of calcium transient upon ZnO NPs exposure was further confirmed in hiPSC-CMs. These data demonstrate that ZnO NPs have acute toxic effects on cardiac electrophysiology and contractile function.

Zhou et al., used an *in vitro* CPB model to study the interaction between THP-1 monocyte-like cells and human neonatal dermal microvascular endothelial cells (HNDMVECs). Sheared THP-1 cells secreted more abundant IL-8 which upregulated vascular cell adhesion molecule 1 (VCAM-1) and intercellular adhesion molecule 1 (ICAM-1) in HNDMVECs and promoted THP-1 cells adhesion to endothelial monolayer and may facilitate transmigration through endothelium. These data elicit a new mechanism of post-CPB inflammation.

Large animal models, such as pigs, are essential for translational cardiovascular research as they more closely resemble human anatomy, physiology, and function. Ascione et al., investigated the feasibility, safety and efficacy of hCD16^+^ monocytes for the treatment of myocardial infarction (MI) in a pig model. Cardiac magnetic resonance analysis suggested that although LVEF did not differ significantly between groups, CD16^+^ monocyte administration resulted in 14.5 g scar reduction (from 25.45 ± 8.24 to 10.8 ± 3.4 gr; −55%) as compared to 6.4 g scar reduction (from 18.83 ± 5.06 to 12.4 ± 3.9 gr; −30%) in the control group 30 days after treatment. In addition, higher tissue levels of neo-angiogenesis, myofibroblast and IL-6 and lower levels of TGF-β were observed in the hCD16^+^ monocytes treated group. These data demonstrate that the use of hCD16^+^ monocytes in acute MI is feasible, safe, and associated with reduced LV scar size, increased neo-angiogenesis, myofibroblasts, and IL-6.

In addition to basic and translation studies, three original articles reported clinical findings. Zhu et al., identified eight differentially expressed genes related to iron metabolism. These genes are mainly involved in the cellular stress response. A logistic regression model based on these genes achieved an AUC of 0.64–0.65 in the diagnosis of coronary heart disease, indicating these genes may have diagnostic potential for coronary heart disease.

Wang et al., examined the data from the USA National Health and Nutrition Examination Survey (NHANES, 2017- 2020.03) in adults aged ≥40 years and explored the association between serum transferrin saturation levels and heart failure. They found that participants with heart failure had significantly lower serum transferrin saturation levels compared to those without heart failure. After fully adjusting for potential confounders, weighted multiple logistic regression models revealed a 2.6% reduced in the risk of heart failure when each unit of serum transferrin saturation level increased. These findings suggest a negative association between serum transferrin saturation levels and heart failure among middle-aged and older adults in the United States.

Liu et al., assessed the value of stress hyperglycemia ratio in determining outcomes in patients with atrial fibrillation in intensive care unit. Among patients with critical atrial fibrillation, those with the highest stress hyperglycemia ratio quartiles exhibited an increased risk of 365-day all-cause mortality (HR: 1.32, 95% CI = 1.06–1.65). Notably, in subgroup analyses, the prognostic value of atrial fibrillation was particularly pronounced in patients with comorbid condition, hypertension. This highlights a positive association between stress hyperglycemia ratio caused by acute glycemic dysregulation and all-cause mortality in critically ill patients with atrial fibrillation.

In summary, this Research Topic provides an additional facet to understanding causes of cardiac disorders, as well as put forth potential therapeutic interventions targeting the dysfunctional myocardium. Ensuing research is crucial to close the gap between existing knowledge with the mechanisms highlighted in this Research Topic as well as further validate the diagnostic and prognostic tools described.

We would like to thank all the authors for their submissions to this Research Topic. We also thank all the reviewers for dedicating their time and helping ensure the quality of the submitted papers, and, last but not least, the staff at the editorial office of Frontiers in Cardiovascular Medicine for their efficient assistance.

